# Efficacy of pre-exercise low-level laser therapy on isokinetic muscle performance in individuals with type 2 diabetes mellitus: study protocol for a randomized controlled trial

**DOI:** 10.1186/1745-6215-15-116

**Published:** 2014-04-09

**Authors:** Cid André Fidelis de Paula Gomes, Ernesto Cesar Pinto Leal-Junior, Daniela Aparecida Biasotto-Gonzalez, Yasmin El-Hage, Fabiano Politti, Tabajara de Oliveira Gonzalez, Almir Vieira Dibai-Filho, Adriano Rodrigues de Oliveira, Marcelo Frigero, Fernanda Colella Antonialli, Adriane Aver Vanin, Paulo de Tarso Camillo de Carvalho

**Affiliations:** 1 Postgraduate Program in Biophotonics Applied to Health Sciences, Universidade Nove de Julho (UNINOVE), Av. Dr. Adolfo Pinto, 109. Água Branca, São Paulo, SP 05001-100, Brazil; 2 Department of Physical Therapy, Universidade Nove de Julho (UNINOVE), Av. Dr. Adolfo Pinto, 109. Água Branca, São Paulo, SP 05001-100, Brazil; 3 Postgraduate Program in Rehabilitation Sciences, Universidade Nove de Julho (UNINOVE), Av. Dr. Adolfo Pinto, 109. Água Branca, São Paulo, SP 05001-100, Brazil; 4 Postgraduate Program in Rehabilitation and Functional Performance, University of São Paulo, Ribeirão Preto, v. Bandeirantes, 3900 - Curso de Fisioterapia/Casa 2, Monte Alegre, São Paulo 14049-900, Brazil

**Keywords:** Diabetes Mellitus, Low-Level Laser Therapy, Muscle, Skeletal, Quadriceps Muscle, Physical Therapy Modalities

## Abstract

**Background:**

Type 2 diabetes, also known non-insulin-dependent diabetes, is the most prevalent type of the disease and involves defects in the secretion and action of insulin. The aim of the proposed study is to evaluate the efficacy of pre-exercise low-level laser therapy (LLLT) on muscle performance of the quadriceps femoris in individuals with type 2 diabetes.

**Methods/Design:**

A double-blind, randomized, controlled clinical trial will be carried out in two treatment phases. In the first phase, quadriceps muscle performance will be evaluated using an isokinetic dynamometer and the levels of creatine kinase and lactate dehydrogenase (biochemical markers of muscle damage) will be determined. The participants will then be allocated to four LLLT groups through a randomization process using opaque envelopes: Group A (4 Joules), Group B (6 Joules), Group C (8 Joules) and Group D (0 Joules; placebo). Following the administration of LLLT, the participants will be submitted to an isokinetic eccentric muscle fatigue protocol involving the quadriceps muscle bilaterally. Muscle performance and biochemical markers of muscle damage will be evaluated again immediately after as well as 24 and 48 hours after the experimental protocol. One week after the last evaluation the second phase will begin, during which Groups A, B and C will receive the LLLT protocol that achieved the best muscle performance in phase 1 for a period of 4 weeks. At the end of this period, muscle performance will be evaluated again. The protocol for this study is registered with the World Health Organization under Universal Trial Number U1111-1146-7109.

**Discussion:**

The purpose of this randomized clinical trial is to evaluate the efficacy of pre-exercise LLLT on the performance of the quadriceps muscle (peak torque, total muscle work, maximum power and fatigue index – normalized by body mass) in individuals with DM-2. The study will support the practice of evidence-based to the use of LLLT in improving muscle performance in Individuals with DM-2. Data will be published after the study is completed.

## Background

Diabetes mellitus (DM) is a heterogeneous group of metabolic disorders characterized by hyperglycemia stemming from defects in the secretion and/or action of insulin. Type 2 DM (DM-2), also known non-insulin-dependent diabetes, accounts for more than 90% of cases and is characterized by insulin resistance and a reduction in insulin-producing β cells in the pancreas as a result of interactions among genetic polymorphisms, obesity, a sedentary lifestyle and the ageing process [1,2].

Diet, drug therapy and physical exercise are highly recommended for weight loss among individuals with DM-2 to improve blood sugar control and avoid the loss of muscle mass [3]. Cardiovascular and strength training are recommended to enhance physical fitness, muscle mass and strength in these individuals [4]. Training programs founded on resistance exercises are considered safe and well tolerated by individuals with DM-2, including elderly individuals [5]. Indeed, resistance training is more efficacious than moderate weight loss for improvements in glycated hemoglobin, which is a commonly employed reference to determine the degree of blood sugar control in individuals with DM [5,6].

Muscle quality is reported to be significantly lower in patients with DM-2 than individuals without this disease [7,8]. Thus, such patients often encounter difficulties when performing exercise programs which increases the risk of diminished physical function and the occurrence of comorbidities and even death [9-11]. Patients with DM-2 seem to activate a limited number of the motor units responsible for muscle contractions, which may be related to the denervation of muscle fibers and/or the increase in intramuscular fat tissue [12].

Low-level laser therapy (LLLT) is a recent option for enhancing muscle performance and is founded on the application of light in the range of 1 to 500 mW on a given region of the body for the promotion of tissue regeneration, the modulation of inflammation, pain relief [13] and improvements in muscle fatigue. LLLT is reported to optimize muscle performance in both animals [14-17] and humans [18,19]. LLLT also improves levels of biological markers directly related to the recovery of the musculoskeletal system when applied before [19], during [20] or after [21] the execution of physical exercise. This therapeutic modality seems to improve muscle performance via the energy metabolism in cells by stimulating photochemical events and enhancing mitochondrial function in muscle cells [22]. The structural changes in the mitochondria (size) promoted by LLLT lead to an improvement in cell respiration and the formation of adenosine triphosphate, which provides energy to the cells [23]. LLLT may be useful in individuals with DM-2, allowing improvements in muscle fatigue and the potentiation of muscle function, with a consequent improvement in exercise capacity and a reduction in the emergence of comorbidities such as diabetic neuropathy.

The hypothesis of the proposed study is that pre-exercise LLLT is capable of improving eccentric muscle performance in individuals with DM-2. If this improvement is confirmed, the use of LLLT can favor the execution of physical exercise and activities of daily living among such patients and avoid the emergence of comorbidities that are common to this disease. LLLT could be widely used in the clinical setting and become an easy-to-administer treatment strategy of considerable value. Moreover, the scientific community is in need of further clinical trials on LLLT involving human subjects for the definition of application parameters, mechanisms of action and the long-term effect on skeletal muscle performance.

The aim of the proposed study is to evaluate the efficacy of pre-exercise LLLT on the performance of the quadriceps femoris (peak torque, total muscle work, maximum power and fatigue index normalized by body mass) in individuals with DM-2.

## Methods/Design

### Overview of research design

A double-blind, randomized, controlled trial will be carried out. The participants will be allocated to four LLLT groups through a randomization process using opaque envelopes: Group A (4 Joules), Group B (6 Joules), Group C (8 Joules) and Group D (0 Joules; placebo). Evaluations will be performed using multidimensional questionnaire, self-perceived fatigue (visual analog scale for fatigue (VAS-F) and Item 2.2 on the WHOQOL-100), Lower Extremity Functional Scale (LEFS), Diabetes Quality of Life (DQOL) measure, Problem Areas in Diabetes (PAID) scale, WHOQOL-BREF, Patient Specific Functional Scale (PSFS), functional evaluations (isokinetic dynamometer, sit-and-stand test, timed up-and-go test (TUGT), functional reach test (FRT)) and blood collection for the assessment of markers of muscle damage and the capillary glycemia test.

The study will be divided into six evaluations and two treatment phases to which all individuals will be submitted.

#### Evaluation 1

After screening, individuals who meet the eligibility criteria will undergo an initial evaluation involving the multidimensional questionnaire, LEFS, DQOL, PAID, WHOQOL-BREF, PSFS, sit-and-stand test, TUGT and FRT. On a separate day, scheduled based on the availability of the individuals, the blood collection, capillary glycemia test and isokinetic dynamometry (maximum voluntary isometric contraction (MVIC) of the quadriceps muscle) will be performed.

#### Treatment phase 1

Immediately after randomization, treatment will be performed with the administration of the LLLT protocol for the respective groups on the quadriceps muscle bilaterally (alternating legs).

#### Evaluation 2

Immediately following LLLT, the groups will perform the isokinetic eccentric muscle fatigue protocol for the quadriceps muscle bilaterally (alternating legs).

#### Evaluation 3

Immediately after the exercise protocol, blood collection, the capillary glycemia test and isokinetic dynamometry (MVIC of the quadriceps muscle bilaterally (alternating legs)) will be performed.

#### Evaluation 4

Twenty-four hours after the exercise protocol, blood collection, the capillary glycemia test and isokinetic dynamometry (MVIC of the quadriceps muscle bilaterally (alternating legs)) will be performed.

#### Evaluation 5

Forty-eight hours after the exercise protocol, blood collection, the capillary glycemia test and isokinetic dynamometry (MVIC of the quadriceps muscle bilaterally (alternating legs)) will be performed.

#### Treatment phase 2

One week after Evaluation 5, Groups A, B and C will receive the LLLT protocol that achieved the best muscle performance in phase 1 and Group D will receive placebo LLLT three sessions per week for a period of 4 weeks (total of 12 sessions).

#### Evaluation 6

Immediately after the 12th session, evaluations will be performed using the LEFS, DQOL, PAID, WHOQOL-BREF, PSFS, sit-and-stand test, TUGT, FRT and MVIC of the quadriceps muscle bilaterally (alternating legs) (Additional files 1 and 2).

### Blinding

The participants will be blinded to the allocation to the different groups. One researcher will be in charge of the randomization process and will program the laser device based on the results of the randomization. A second researcher will perform the LLLT and will be blinded to the doses and respective groups. These researchers will not participate in any of the evaluation phases, which will be performed by a third researcher blinded to the allocation of the subjects to the different groups. When blood collection is involved, the evaluations will be performed by an experienced, trained nurse, who will only participate in these evaluations. The statistician will be blinded to the group allocation until completion of the statistical analyses.

### Inclusion criteria

Diagnosis of DM-2 confirmed by the participant’s physician; age between 30 and 70 years; either gender; sufficient cognitive level for understanding the procedures and following the instructions; and agreement to participate in the study by signing of a statement of informed consent after receiving clarifications regarding the objectives.

### Exclusion criteria

Diagnosis of cardiovascular disorder; hepatic diseases; pernicious anemia; diagnosis of diabetic neuropathy; occurrence of skin lesion or local infection over the quadriceps muscle; history of skeletal muscle injury in previous six months or during the execution of the study; inability to perform exercise protocol or undergo evaluations in a satisfactory manner; current practice of regular physical activity; and chronic joint disease in lower limbs. Exclusion criteria will be determined by access to medical history of individuals.

### Ethical considerations

The proposed study received approval from the local Human Research Ethics Committee under process number n° 164549 dated 5 December 2012. The study will be conducted in compliance with the norms that regulate research involving human subjects contained in Resolution n° 196/97 of the Brazilian National Health Council. The study is registered with the World Health Organization under Universal Trial Number U1111-1146-7109.

All participants will be informed regarding the objectives and procedures of the study and will be asked to sign a statement of informed consent agreeing to participate.

### Procedures

#### Questionnaires

At the beginning (Evaluation 1) and end (Evaluation 6) of the study, the following questionnaires will be employed:

– A multidimensional questionnaire drafted by the researchers addressing clinical and socio-demographic variables (gender, weight, height, age, marital status, time elapsed since the diagnosis of DM-2, schooling, other diagnosed conditions, medications in use and dose of medications in use).

– LEFS: developed in English to evaluate musculoskeletal disorders of the lower limbs [24], the LEFS consists of 20 items addressing different activities. Each item has a five-option response scale, which is scored as follows: 0 (extreme difficulty or unable to perform); 1 (quite a bit of difficulty); 2 (moderate difficulty); 3 (a little bit of difficulty); and 4 (no difficulty). The maximum score is 80 points, with lower scores denoting greater difficulty [25]. The LEFS has been adapted and validated for Portuguese, with Cronbach’s alpha = 0.95 [25].

– DQOL: this questionnaire has 46 multiple-choice items for the evaluation of quality of life among individuals with diabetes and is made up of four subscales: satisfaction, impact, social/occupational worries and worries related specifically to diabetes. The response options are organized on a five-point Likert scale: 1 = very satisfied; 2 = quite satisfied; 3 = moderately satisfied; 4 = somewhat dissatisfied; and 5 = completely dissatisfied. For the impact and worries subscales, the items are scored as follows: 1 = never; 2 = rarely; 3 = sometimes; 4 = often; and 5 = always. Lower scores denote a better quality of life [26]. The DQOL has been translated and validated for Portuguese and demonstrates adequate reliability and validity for patients with DM-2 [26].

– PAID: questionnaire composed of 20 items on the emotional state of individuals with DM (types 1 and 2). Each item has a five-point Likert response scale: 0 = not a problem; 1 = small problem; 2 = moderate problem; 3 = nearly a serious problem; and 4 = serious problem. The score ranges from 0 to 100 points, with higher scores denoting a greater degree of emotional suffering [27]. The Portuguese version will be used in the proposed study, which has a Cronbach’s alpha = 0.93 for patients with DM-2 [28].

– WHOQOL-BREF: this questionnaire is the short form of the WHOQOL-100, which was developed by the Quality of Life workgroup of the World Health Organization and has previously been used on individuals with DM-2 [29]. The questionnaire is made up of 26 items, two on global and general health and the others distributed among four subscales: physical health, psychological health, social relations and environment. Each item is scored using a Likert scale (1 to 5 points). The results are transformed into a linear scale (0 to 100), with higher scores denoting a better quality of life [30]. The Brazilian version of the WHOQOL-BREF has demonstrated satisfactory internal consistency for both the items and subscales and is a useful tool for the evaluation of quality of life in the Brazilian population [31]. This questionnaire will be administered during Evaluations 1 and 6 and will also be administered by telephone 3 and 6 months after Evaluation 6.

– PSFS: the aim of this self-report scale is to evaluate functional changes, especially those related to the musculoskeletal system. This scale can be used for any part of the body in individuals with different degrees of functionality and different diseases. Function is self-rated on five activities the individual considers important and is having difficulty performing or is unable to perform. Each activity identified by the individual is rated using an 11-point Likert scale (0–11 points), for which higher scores denote greater functionality [32].

– Self-perceived fatigue: subjective fatigue following the MVIC and isokinetic eccentric exercise protocol of the quadriceps muscle will be determined using the VAS-F and item F2.2 on the WHOQOL-100 (physical subscale). For the VAS-F, a color scale measuring 50 cm in length will be printed on a piece of paper, beginning with light blue (minimum) and proceeding to intense red (maximum). The individual will mark their subjective evaluation of muscle fatigue on the scale. On the other side of the card, the examiner will have access to a numeric scale ranging from 0 (minimum) to 10 (maximum) and record the value corresponding to the subject’s mark on the opposite side. Only the examiner will have access to the numeric scale [33,34].

All questionnaires will be administered in a reserved room and are to be filled out completely. If an individual refuses or is unable to cooperate, Examiner 2 will mark “NR” (no record) in capital letters on the exam. To avoid hurried responses from the participants, no time constraints will be applied.

#### Functional assessment tests

– Sit-and-stand test: a standardized chair will be used for all individuals, with an adjustable height to maintain the knees flexed at 90° and no arm rests. Sit-and-stand was chosen due the fact that this movement is one of the activities of daily living of greater demand in terms of mechanical aspects [35]. The individual will be instructed to sit down and stand up from the chair five times. The execution time will be recorded with the aid of a stopwatch (OREGON^©^ - Oregon Scientific Brasil LTDA – http://www.oregonscientific.com - Avenida Ibirapuera, 2907 – São Paulo/SP. Phone: 55 11 50952329).

– TUGT: this test has been used to evaluate function in individuals with DM-2 [36]. The TUGT measures functional mobility based on the time required to stand up from a chair, walk three meters, turn around, walk back to the chair and sit down again. The test will begin with the verbal command “go” and will end when the participant is once again seated in the chair. The execution time will be recorded with the aid of a stopwatch (OREGON^©^). The test will be performed once for the participant to become familiarized with the procedure and the second trial will be used to record the execution time. A standardized chair will be used for all individuals, with an adjustable height to maintain the knees flexed at 90° and no arm rests [37]. The following are the reference parameters for this test: 10 seconds for healthy, independent adults with no risk of falls; 11 to 20 seconds for elderly individuals with disability or frailty; and greater than 20 seconds for elderly individuals with important mobility impairment and a risk of falls [38].

– FRT: this test measures the maximum distance an individual can reach forward beyond the length of his/her arm while maintaining a fixed standing base. The FRT has been used to determine movement strategies in patients with DM [39]. A reach less than 15 cm indicates frailty and an eminent risk of falls [40]. A metric tape will be attached to the wall parallel to the ground at the height of the individual’s acromion. The participant will be barefoot with the feet aligned and standing next to the beginning of the tape measure, with the wrists in a neutral position, elbows extended and the shoulder flexed at 90°. The individual will be instructed to lean forward without touching the tape. The movement of the wrist over the tape will be read three times and the mean will be used for the analysis [39].

#### Blood collection

Blood samples (10 ml) will be collected through the antecubital vein by an experienced nurse. One hour after collection, the samples will be centrifuged at 3,000 rpm for 20 minutes. Pipettes will be used to transport the serum to Eppendorf® tubes (Eppendorf, Hamburg, Germany) which will be stored at -80°Cuntil analysis. Enzyme activity of creatine kinase (CK; CK-MB, CK-MM and CK-BB) and lactate dehydrogenase (LDH; LHD1,2,3,4,5) will be analyzed. The biochemical markers of muscle damage will be analyzed in a clinical laboratory [41,42].

Capillary glycemia will be monitored by the individuals themselves. The use of the equipment will be explained prior to the procedure. Blood will be taken from the palm side of the tip of the third finger on the right hand using a digital blood sugar meter (Accu-Chek® Performa nano; Roche Diagnosis (9115 Hague Road, Indianapolis, United States) and lancet (Accu-Chek® multiclix; Roche Diagnostics (9115 Hague Road, Indianapolis, United States)). The lancet will be standardized at the deepest penetration (number 5). The measure of capillary glycemia and fast, highly precise digital monitors is currently considered the gold standard for at-home blood sugar control in patients with DM and will be used in the present study to determine the oscillation in blood sugar in the different phases of the study [43].

#### Isokinetic dynamometry

The Biodex System 3 Pro isokinetic dynamometer (Biodex Medical System System 4, Biodex Medical Systems, Inc., Shirley, NY, USA) will be used for the eccentric fatigue protocol and MVIC of the quadriceps muscle bilaterally (alternating sides). This device is considered reliable and reproducible for muscle evaluations [44] and has been used on patients with different diseases such as heart failure [45], osteoarthritis [46] and spinal cord injury [47], as well as on different populations ranging from children [46] to the elderly [47-49]. In the proposed study, peak torque (maximum generation of force by the muscle) and the Muscle Fatigue Index [50,51] will be analyzed.

The volunteer will first perform three 60-second sets of active stretching of the quadriceps muscle bilaterally, followed by warm up consisting of pedaling a stationary bike (Inbramed®, Inbramed, Porto Alegre, RS, Brazil) at 100 rpm for 5 minutes without load. The volunteer will then be positioned on the isokinetic dynamometer (previously calibrated following the instructions in the manual), duly aligned and stabilized by straps to avoid compensatory movements. The rotation axis of the dynamometer will be aligned with the movement axis of the knee being evaluated at the level of the lateral epicondyle of the femur. Chair height and base, distance of the support, accessory level and base of the dynamometer will be adjusted to the needs of each individual. During the test, the volunteer will be instructed to cross his/her arms over the chest and the axis of the dynamometer will be positioned parallel to the center of the knee.

**Figure 1:**
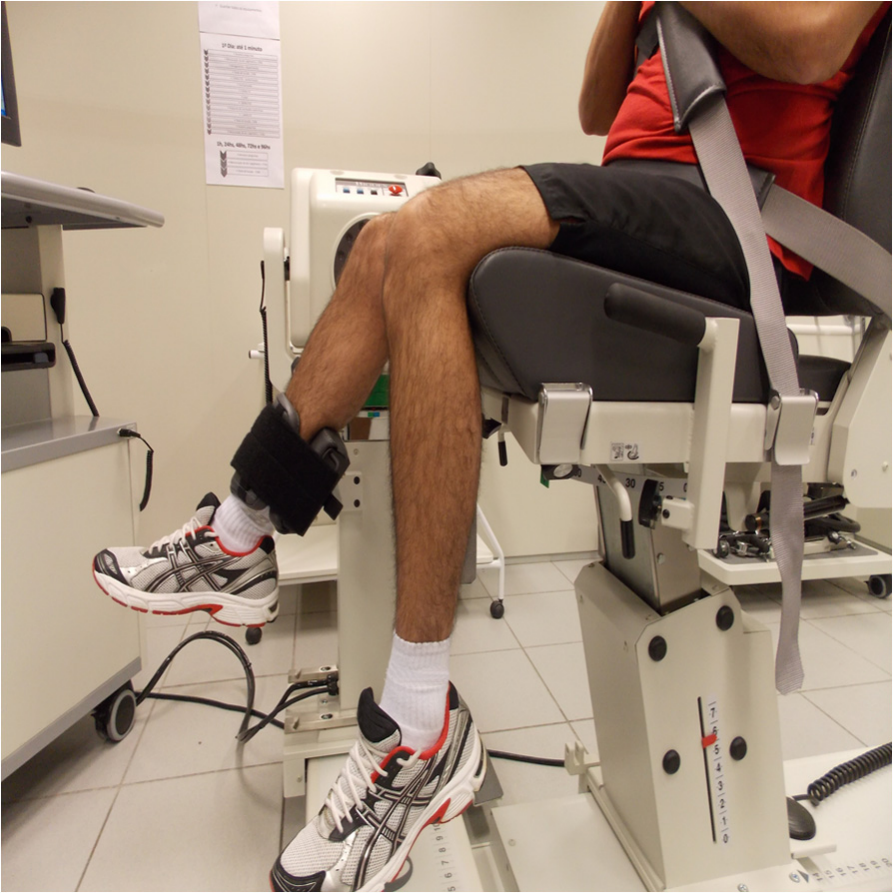
Realization of isokinetic dynamometry maximum voluntary isometric contraction (MVIC) of the quadriceps muscle.

MVIC will consist of three 5-second isometric contractions of the knee extensors (Figure 1). The greatest torque of the three contractions (peak torque) will be used in the statistical analysis. This variable was chosen based on the fact that it reflects the maximum generation of force by the muscle [52]. The instructions will be given first and the volunteers will receive verbal encouragement during the MVIC.

**Figure 2:**
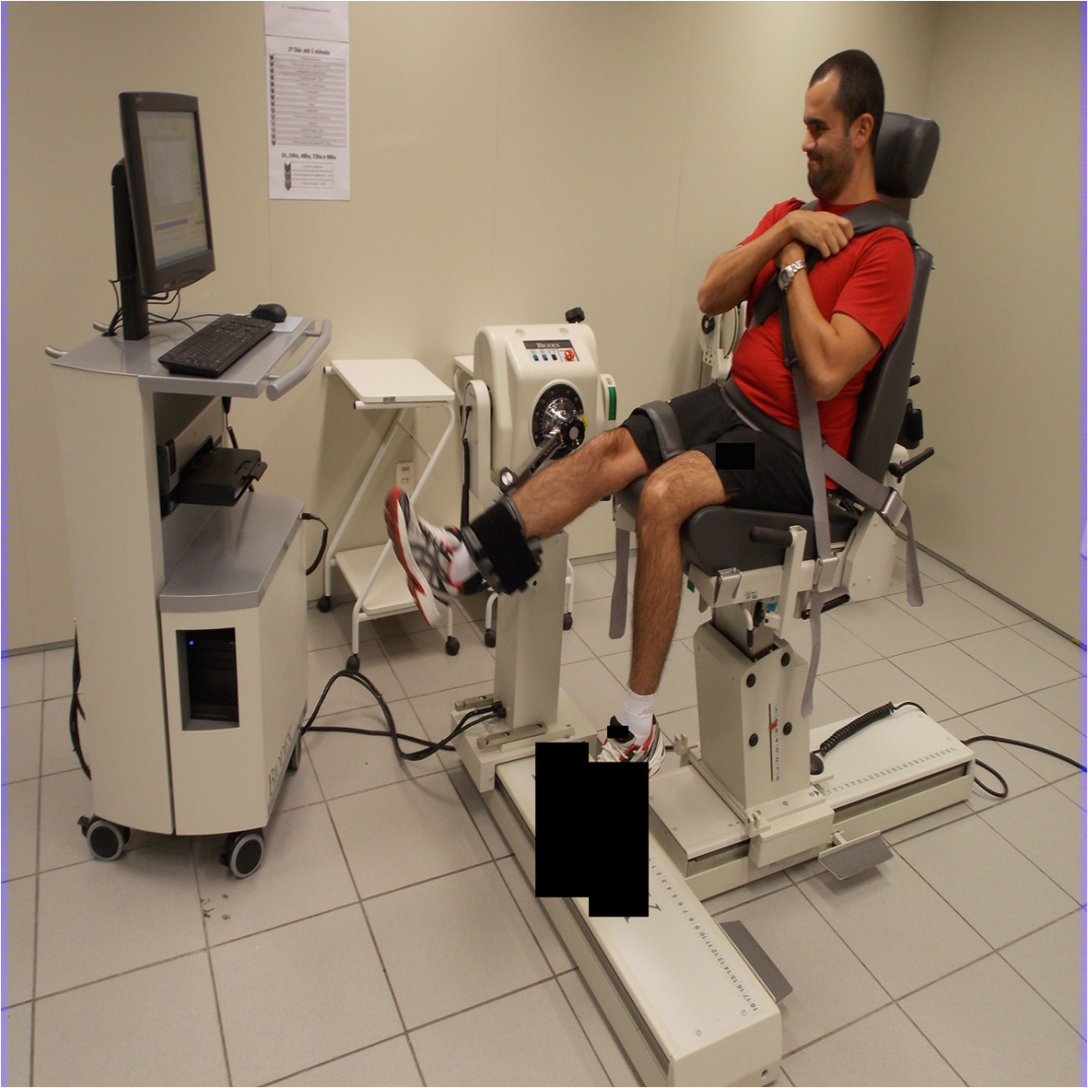
Realization of the isokinetic eccentric fatigue protocol for the quadriceps muscle.

The volunteers will perform the isokinetic eccentric fatigue protocol for the quadriceps muscle bilaterally (Figure 2), which will consist of 75 isokinetic eccentric contractions of the knee extensors (5 sets of 15 repetitions with a 30-second rest period between sets) at a velocity of 60°.sec^-1^ in both the eccentric and concentric phases, with a 60° range of motion (between 90° and 30° of knee flexion) for each leg. At each contraction, the dynamometer will automatically (passively) position the knee at 30° and will then flex the knee until reaching 90°. The volunteers will be instructed to resist the knee flexion imposed by the dynamometer with maximum force. The instructions will be given first and the volunteers will receive verbal encouragement throughout the exercise. This protocol has proven to be efficient and reliable for exercise-induced muscle damage [19].

The order on both the MVIC and isokinetic eccentric fatigue protocol for the quadriceps muscles will be determined randomly by lots using sealed, opaque envelopes.

#### Low-level laser therapy protocol

A five-diode cluster laser device (810 nm and 200 mW for each diode) (THOR Photomedicine®, Thor Photomedicine, Chesham, UK) will be used for LLLT. For the placebo group, a laser device that produces the same characteristics (cluster) will be used, but will not emit Joules. The LLLT protocol will be performed with the cluster in direct contact with the skin at six different sites over the quadriceps muscle (2 medial, 2 lateral and 2 central), totaling 30 points on each leg. The volunteers will be distributed in the following groups:

– Group A – 4 Joules per point (irradiation time: 20 seconds), 120 Joules of total energy irradiated over the muscle with 120 seconds of total irradiation time.

– Group B – 6 Joules per point (irradiation time: 30 seconds), 180 Joules of total energy irradiated over the muscle with 180 seconds of total irradiation time.

– Group C – 8 Joules per point (irradiation time: 40 seconds), 240 Joules of total energy irradiated over the muscle with 240 seconds of total irradiation time.

– Group D – 0 Joules per point (“irradiation” time: 40 seconds), 0 Joules of total energy irradiated over the muscle with 240 seconds of total “irradiation” time.

The order of the LLLT protocol on the quadriceps muscles will be determined randomly by lots using sealed, opaque envelopes.

#### Data analysis

Peak torque determined by the isokinetic dynamometer will be the primary outcome and CK and LDH activity determined from the blood samples will be the secondary outcomes. The Shapiro-Wilk test will be used to test the data with regard to Gaussian distribution. Data will be expressed as mean and standard deviation values. Two-way repeated-measures analysis of variance with the *post hoc* Bonferroni test will be used for inter-group and intra-group comparisons. The level of significance will be set to 5% (*P* < 0.05) for all comparisons. Statistical analysis will be performed using the SPSS program, version 13.0 (Chicago, IL, USA).

## Discussion

The purpose of this randomized clinical trial is to evaluate the efficacy of pre-exercise LLLT on the performance of the quadriceps muscle (peak torque, total muscle work, maximum power and fatigue index – normalized by body mass) in individuals with DM-2. The study will support the practice of evidence-based to the use of LLLT in improving muscle performance in Individuals with DM-2. Data will be published after the study is completed.

## Trial status

Patient recruitment is currently underway.

## Abbreviations

CK: creatine kinase; DM: diabetes mellitus; DM-2: type 2 diabetes mellitus; DQOL: Diabetes Quality of Life; FRT: functional reach test; LDH: lactate dehydrogenase; LEFS: Lower Extremity Functional Scale; LLLT: low-level laser therapy; MVIC: maximum voluntary isometric contraction; NR: no record; PAID: Problem Areas in Diabetes; PSFS: Patient Specific Functional Scale; TUGT: timed up-and-go test; VAS-F: visual analog scale for fatigue.

## Competing interests

Professor Ernesto Cesar Pinto Leal-Junior receives research support from Multi Radiance Medical (Solon, OH - USA), a laser device manufacturer. The remaining authors declare that they have no competing interests.

## Authors’ contributions

CAFdPG: conception and design, data collection and analysis, manuscript writing and final approval of the manuscript. ECPLJ: conception and design, financial support, manuscript writing, final approval of manuscript. DABG: conception and design, financial support, manuscript writing, final approval of manuscript. YEH: data collection and analysis, critical revision and final approval of the manuscript. FP: data collection and analysis, critical revision and final approval of the manuscript. TdOG: data collection and analysis, critical revision and final approval of the manuscript. AVDF: data collection and analysis, critical revision and final approval of the manuscript. ARdO: data collection and analysis, critical revision and final approval of the manuscript. MF: data collection and analysis, critical revision and final approval of the manuscript. FCA: data collection and analysis, critical revision and final approval of the manuscript. AAV: data collection and analysis, critical revision and final approval of the manuscript. PdTCdC: conception and design, financial support, manuscript writing, final approval of manuscript. All authors read and approved the final manuscript.

## Supplementary Material

Additional file 1 Flowchart phase 1. Click here for file

Additional file 2 Flowchart phase 2. Click here for file
