# Supplementary figures and images for: Efficacy of pre-exercise low-level laser therapy on isokinetic muscle performance in individuals with type 2 diabetes mellitus: study protocol for a randomized controlled trial
Source: Trials. 2014 Apr 9;15:116. doi: 10.1186/1745-6215-15-116 (PMC4021637; doi:10.1186/1745-6215-15-116)

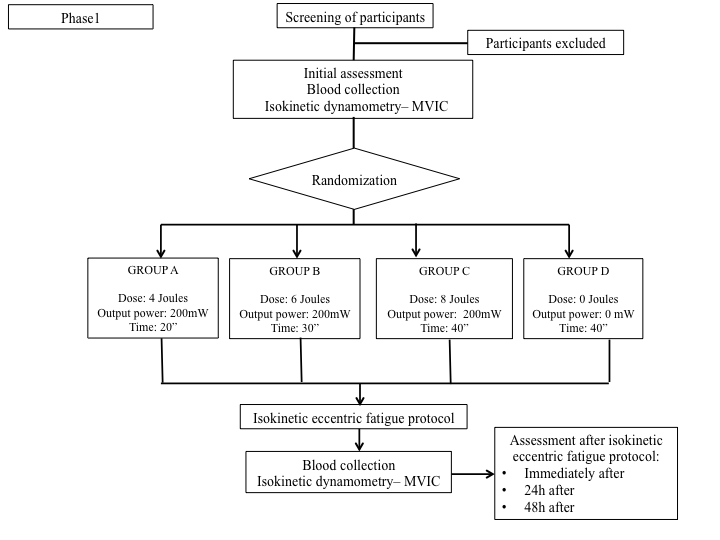

Supplement: Additional file 1 — Flowchart phase 1. [file 1745-6215-15-116-S1.tiff]

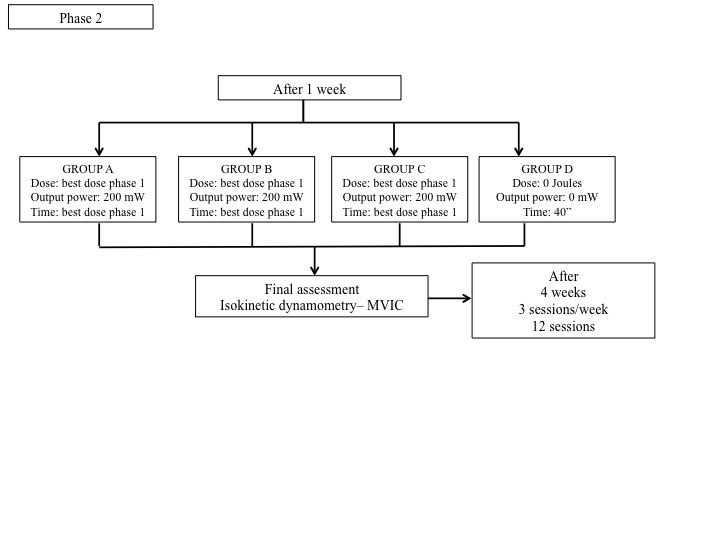

Supplement: Additional file 2 — Flowchart phase 2. [file 1745-6215-15-116-S2.tiff]
